# The effect of baseline versus early glucocorticoid use on immune checkpoint inhibitor efficacy in patients with advanced NSCLC

**DOI:** 10.3389/fonc.2025.1533556

**Published:** 2025-01-23

**Authors:** Yifan Wang, Jianying Zhou, Simin Peng, Zhao Cui, Weiqi Wang, Wenqin Zeng, Tingting Qiu, Zhentian Liu

**Affiliations:** ^1^ Department of Thoracic Medical Oncology, Jiangxi Provincial Cancer Hospital, Nanchang, Jiangxi, China; ^2^ Jiangxi Medical College, Nanchang University, Nanchang, Jiangxi, China

**Keywords:** glucocorticoids, immune checkpoint inhibitors, efficacy, timing of use, NEUT%/(CD4+/CD8+), non-small cell lung cancer

## Abstract

**Purpose:**

This study aims to investigate the specific effects of glucocorticoids (GC) on the efficacy of immune checkpoint inhibitors (ICIs), and whether this effect is influenced by the timing and dosage of GC administration. Changes in the neutrophil percentage and the helper/suppressor T lymphocyte ratio [NEUT %/(CD4+/CD8+)] during GC administration were monitored.

**Methods:**

The clinical results of 130 patients with advanced non-small cell lung cancer (NSCLC) treated with ICIs were analyzed and compared with those of patients who did not use GC. Cox proportional hazards regression model and Logistic regression analysis were used to analyze the factors affecting ORR and PFS, and t test was used to analyze the changes of NEUT %/(CD4 +/CD8 +) during GC use.

**Results:**

Multivariate Logistic analysis showed that GC use was associated with a higher ORR in 130 patients treated with ICIs [HR = 3.07,95% CI (1.31-7.21), P = 0.010]. Univariate Cox analysis showed that GC use was not significantly correlated with PFS [HR = 0.926,95% CI (0.603-1.420), P = 0.710]. Patients who used GC during the baseline period of ICIs treatment had a higher ORR than those who used GC at the early stage of ICIs treatment (65.4% vs 30.8%, p = 0.024). Multivariate Cox analysis showed that GC use had longer PFS [HR = 0.37,95% CI (0.17-0.78), p = 0.009]. The timing of GC use was different, and there was a difference in NEUT %/(CD4 +/CD8 +) levels before and after treatment. There was no significant difference in ORR and PFS between GC duration and dose.

**Conclusion:**

The use of GC helps to enhance the efficacy of immunotherapy. In particular, GC use during the baseline period leads to higher ORR and PFS, regardless of the dose or duration of GC use. The levels of NEUT %/(CD4+/CD8+) varied depending on the timing of GC administration.

## 1 Introduction

Immune checkpoint inhibitors (ICIs) have become the standard treatment for non-small cell lung cancer (NSCLC), which accounts for 85-90% of all lung cancer cases (1–3). Nevertheless, a limited number of patients exhibit a response to ICIs (4). Despite its widespread use, assessing programmed death ligand-1 (PD-L1) expression remains inadequate for clinical needs (3, 5). The Eastern Cooperative Oncology Group (ECOG) performance status and concurrent use of glucocorticoids (GC) may influence the efficacy of ICIs (6–12).

GC has strong anti-inflammatory, analgesic, and antiemetic properties, making it a common adjunct in oncological therapy (13). However, its impact on T-cell function, count, and immunosuppressive effects may diminish the antitumor efficacy of ICIs (12, 14–17). Prednisone therapy (≥ 10 mg/day) before initiating ICIs in NSCLC patients has been associated with poor prognosis and is closely linked to GC indications (18). Some studies found that baseline GC use does not affect survival outcomes, but they do not account for variables like time and may yield different results after adjustment (19). A meta-analysis of ICIs before and after chemotherapy found that patients receiving dexamethasone had a higher survival rate. These studies suggest that GC timing, dosage, and strategies influence immunotherapy efficacy, though these factors remain underexplored (18–24).

This study assessed the effects of GC administration, either at baseline or early stages, on ICIs efficacy in NSCLC patients. It also explored the relationship between GC timing and patient outcomes, highlighting an association between the NEUT%/(CD4+/CD8+) ratio and clinical outcomes.

## 2 Patients and methods

### 2.1 Study population

**Figure 1:**
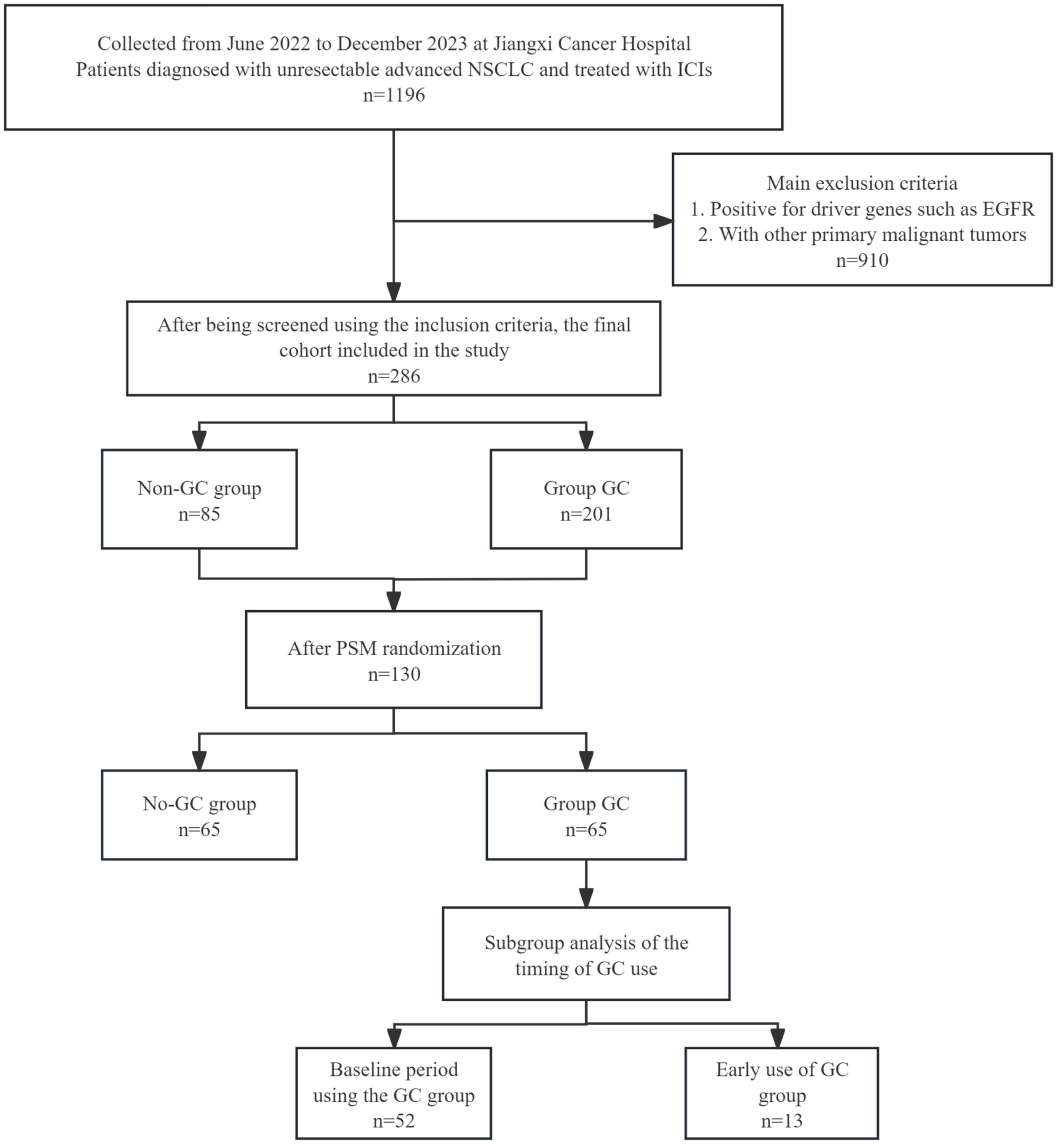
Flowchart of the 286-patient cohort included and the 130 NSCLC patients included in the final analysis. ICIs, immune checkpoint inhibitors; NSCLC, non-small cell lung cancer; PSM, propensity score matching.

This retrospective study included patients with advanced NSCLC who received at least two ICIs treatments at Jiangxi Provincial Cancer Hospital between June and December 2022 (**Figure 1**). It included an analysis of various demographic and clinical features, such as age, sex, and tobacco use history, pathological type, TNM stage, programmed death ligand-1(PD-L1) expression [Tumor Proportion Score (TPS)], ECOG performance status, treatment strategies, lines of therapy, and GC use within one month before and after ICI treatment, indications for GC use [including tumor-related, non-tumor-related reasons, infectious and immune-related adverse events (irAEs), and chemotherapy pretreatment], duration and timing of GC use, antibiotic (ATB) use, proton pump inhibitor (PPI) use, and radiation therapy (RT) use, baseline data, and post-treatment imaging and laboratory assessments every 2 months. Baseline and follow-up imaging (CT, MRI, etc.) and laboratory assessments, which included blood routine and measurements of serum oncological markers, among others were conducted bimonthly post-treatment.

Consistent with established consensus and the GC usage protocols at our center, oral, intramuscular, or intravenous GC administration of ≥ 10 mg prednisone equivalents within 30 days prior to initial ICIs treatment is defined as baseline use; GC administration of ≥ 10 mg prednisone equivalents within 30 days following the initial ICI treatment is defined as early use; and no GC administration, or GC administration of 0 to < 10 mg prednisone equivalents, is defined as no GC use.

The study adhered to the principles of the Declaration of Helsinki throughout its duration and received approval from the Medical Ethics Committee of Jiangxi Cancer Hospital (Approval No. 2024ky167).

### 2.2 Research endpoint

The primary endpoint of the study was the objective response rate (ORR), with progression-free survival (PFS) as the secondary endpoint. Imaging assessments were conducted in accordance with the Response Evaluation Criteria in Solid Tumors (RECIST) version 1.1. The last follow-up was documented on 30 July 2024. The ORR was calculated as the total number of patients with complete response (CR) and partial response (PR), represented as a percentage of all patients. PFS was measured from the start of ICI therapy until the occurrence of disease progression or death.

### 2.3 Statistical analysis

SPSS 27.0 and R 4.4.1 statistical software (www.r-project.org) were used to analyze the data. In order to control the confounding factors, a post-randomized population was constructed.,Propensity score matching (PSM) grouping variable was the use of GC. The independent variables were gender, ECOG score, histological type, TNM stage, treatment strategy, number of treatment lines, brain metastasis, bone metastasis and liver metastasis. The independent variables with p < 0.2 were used to construct the matching model. The caliper value was 0.05, and 1: 1 matching according to the nearest neighbor matching method. The balance test was performed before and after matching in **Table 1** and **Supplementary Table 1**. Descriptive statistics are used to summarize the variables and are expressed in terms of numbers and percentages. X^2^ test score or Fisher exact probability method was used to analyze the relationship between GC medication and clinical characteristics of patients. The survival curve of PFS was drawn by Kaplan-Meier method, and the survival test was performed by Log-rank. The factors affecting ORR and PFS were analyzed by univariate and multivariate Cox risk proportional regression model and binary logistic regression analysis. In the univariate analysis, P < 0.1 and the factors that are likely to affect the outcome according to clinical experience are also included in the analysis. Two independent samples t test was used to analyze the difference of NEUT %/(CD4 +/CD8 +) between GC group and Non-GC group, baseline and early GC before use. Paired sample t test was used to analyze the difference of NEUT %/(CD4 +/CD8 +) before and after GC use at baseline and early stage. All P values were based on the two-sided hypothesis, and P < 0.05 was considered statistically significant.

**Table 1:** Clinical Characteristics of 130 Patients with Advanced NSCLC Receiving ICIs [n (%)].

Characteristics	Classification	Total (n = 130)	Non-GC (n=65 )	GC (n=65)	p
Gender					
	Female	31 (23.85)	12 (18.46)	19 (29.23)	0.150
	Male	99 (76.15)	53 (81.54)	46 (70.77)	
Age					
	≤65	71 (54.62)	33 (50.77)	38 (58.46)	0.378
	>65	59 (45.38)	32 (49.23)	27 (41.54)	
Smoke					
	NO	47 (36.15)	19 (29.23)	28 (43.08)	0.100
	Yes	83 (63.85)	46 (70.77)	37 (56.92)	
ECOG					
	≤1	64 (49.23)	32 (49.23)	32 (49.23)	1.000
	>1	66 (50.77)	33 (50.77)	33 (50.77)	
Histology					
	No Squamous	81 (62.31)	34 (52.31)	47 (72.31)	0.019
	Squamous	49 (37.69)	31 (47.69)	18 (27.69)	
TNM					
	III	32 (24.62)	16 (24.62)	16 (24.62)	1.000
	IV	98 (75.38)	49 (75.38)	49 (75.38)	
PD-L1					
	<1	17 (13.08)	9 (13.85)	8 (12.31)	0.931
	1-49	9 (6.92)	4 (6.15)	5 (7.69)	
	>50	12 (9.23)	5 (7.69)	7 (10.77)	
	Unknown	92 (70.77)	47 (72.31)	45 (69.23)	
Brain					
	NO	106 (81.54)	54 (83.08)	52 (80.00)	0.651
	Yes	24 (18.46)	11 (16.92)	13 (20.00)	
Bone					
	NO	89 (68.46)	48 (73.85)	41 (63.08)	0.186
	Yes	41 (31.54)	17 (26.15)	24 (36.92)	
Liver					
	NO	116 (89.23)	60 (92.31)	56 (86.15)	0.258
	Yes	14 (10.77)	5 (7.69)	9 (13.85)	
Strategy					
	Combination	120 (92.31)	60 (92.31)	60 (92.31)	1.000
	Single	10 (7.69)	5 (7.69)	5 (7.69)	
Line					
	≥Second line	44 (33.85)	24 (36.92)	20 (30.77)	0.458
	First line	86 (66.15)	41 (63.08)	45 (69.23)	

## 3 Results

### 3.1 Patient characteristics

In this study, we assessed 130 patients with advanced NSCLC who were treated with ICIs, with baseline characteristics presented in **Table 1**. Among them, 99 (76.15%) were male, 71 (54.62%) were under 65, and 64 (49.23%) had an ECOG status of 0-1. Most (63.85%) were smokers, and 81 (62.31%) had non-squamous cell carcinoma.98 (75.38%) patients were classified as TNM stage IV. 38 (29.23%) patients had tumor samples analyzed for PD-L1 expression. Immuno-combination therapy was administered to 120 (92.31%) patients, and 86 (66.15%) patients received first-line therapy.

PSM achieved balance across population characteristics. Analyzing clinical data, GC use showed no significant link to age, gender, smoking, ECOG status, TNM stage, PD-L1 levels, brain metastases presence, or treatment lines (p > 0.05), and was significantly associated with histological type (p = 0.019; **Table 1**), possibly due to the high percentage of patients pretreated with pemetrexed chemotherapy in adenocarcinoma.

### 3.2 Impact of GC use on efficacy of ICIs treatment in patients with NSCLC

**Figure 2:**
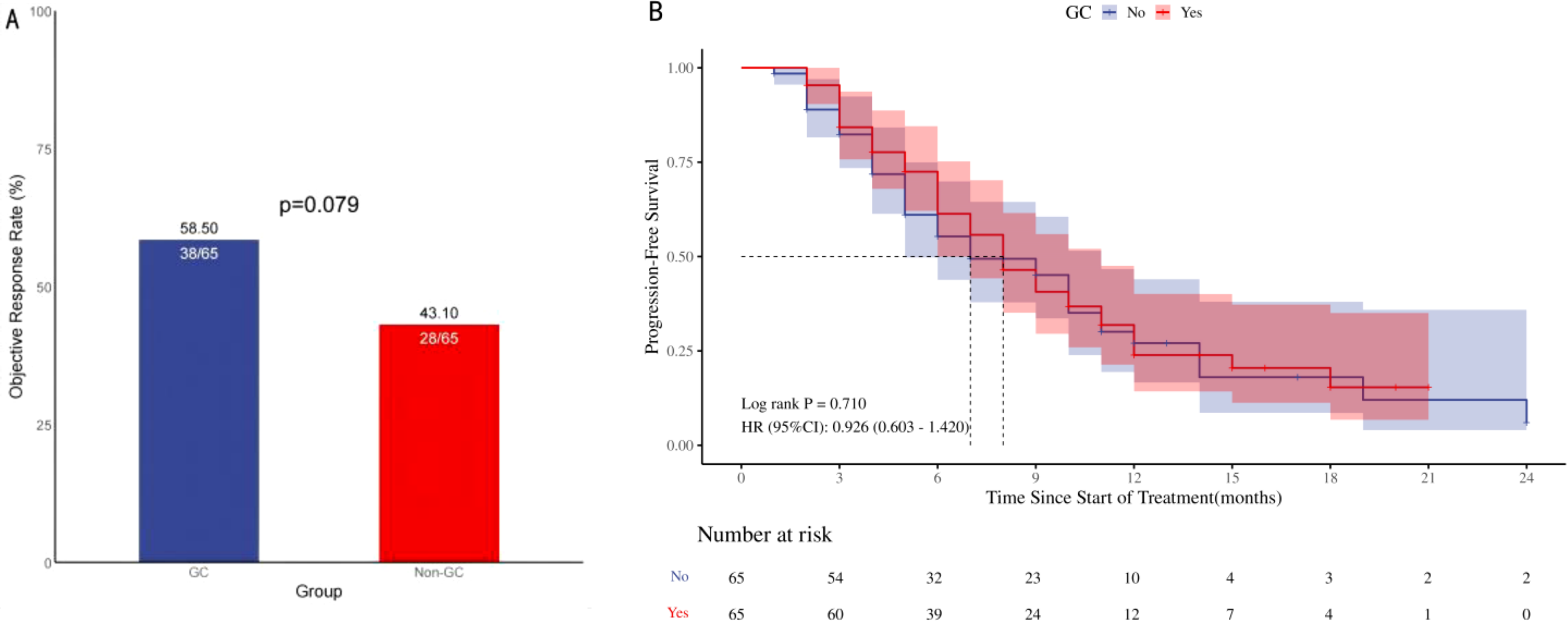
ORR and PFS in 130 patients with advanced NSCLC treated with ICIs. Objective remission rate (**A**; ORR), Progression-free survival (**B**; PFS), ORR and PFS in patients with Advanced NSCLC: Comparison of Patients Treated with <10 mg/d Prednisone versus ≥10 mg/d Prednisone. ORR, Objective remission rate; PFS, Progression-free survival; HR, Hazard ratio.

Individuals administered GC at the commencement of therapy or in the early phase of ICIs treatment exhibited an ORR of 58.5% versus 43.1% (p=0.079; **Figure 2A**), which, however, this difference did not reach statistical significance. Univariate logistic regression analysis revealed no significant difference in the ORR between patients with advanced NSCLC who received GC therapy and those who received ICIs, although a trend approaching significance was observed. The difference was significant in the follow-up multifactorial analysis [HR=3.07, 95% CI [1.31-7.21], p=0.010); **Table 2**, this suggests that the initial analysis may have concealed the true impact of GC use, which was revealed through a regression model that accounted for other confounding variables. ECOG performance status, histological subtype, and the number of treatment lines were also found to be associated with variations in the ORR across the different groups.

**Table 2:** Univariate and multifactorial analysis of ORR in 130 patients with advanced NSCLC treated with ICIs.

Characteristics	Reference	Univariate *HR* (95% *CI*)	p	Multivariate *HR* (95% *CI*)	p
Age	≤65	0.89 (0.45-1.77)	0.737		
Gender	Female	2.73 (1.17-6.41)	0.021	1.66 (0.62-4.49)	0.316
Smoke	NO	1.47 (0.71-3.01)	0.297		
ECOG	≤1	0.50 (0.25-1.01)	0.054	0.37 (0.17-0.84)	0.017
Histology	No Squamous	4.03 (1.88-8.66)	<0.001	4.68 (1.86-11.81)	0.001
TNM	III	0.75 (0.33-1.67)	0.476		
Brain	NO	0.64 (0.26-1.56)	0.325		
Bone	NO	0.77 (0.37-1.62)	0.494		
Liver	NO	0.97 (0.32-2.93)	0.951		
Line	≥Second line	3.28 (1.52-7.06)	0.002	2.54 (1.08-6.01)	0.033
Strategy	Combination	0.62 (0.17-2.32)	0.482		
GC	NO	1.86 (0.93-3.73)	0.081	3.07 (1.31-7.21)	0.010
ATB	NO	0.82 (0.37-1.79)	0.615		
PPIs	NO	1.66 (0.80-3.47)	0.176		
RT	NO	1.41 (0.69-2.86)	0.346		

The comparison of PFS between the two groups did not yield a statistically significant difference [HR=0.926, 95% CI (0.603-1.420), p=0.710]; **Figure 2B**. The survival curves of the two cohorts, as assessed by Kaplan-Meier analysis, showed no evident separation, suggesting a lack of statistically significant variation in survival outcomes. The univariate Cox regression analysis showed that the use of GC did not have a significant impact on PFS among patients with advanced NSCLC undergoing ICIs therapy [HR=0.93, 95% CI (0.60-1.42), p=0.723]; **Table 3**, Conversely, the number of treatment lines was significantly correlated with PFS [HR=3.28, 95% CI (1.52-7.06), p=0.002]. The multivariable analysis indicated that the use of GC was not a significant factor influencing PFS, while the observed differences in survival curves among the groups were primarily due to the variation in the number of treatment lines [HR=0.35, 95% CI (0.21-0.59), p<0.001] and radiation therapy [HR=0.47, 95% CI (0.29-0.78), p=0.003].

**Table 3:** Univariate and multifactorial analysis of PFS in 130 patients with advanced NSCLC treated with ICIs.

Characteristics	Reference	Univariate *HR* (95% *CI*)	p	Multivariate *HR* (95% *CI*)	p
Age	≤65	0.89 (0.57-1.38)	0.607		
Gender	Female	0.53 (0.33-0.83)	0.006	1.05 (0.47-2.33)	0.908
Smoke	NO	0.66 (0.43-1.01)	0.057	0.74 (0.37-1.49)	0.404
ECOG	≤1	0.92 (0.60-1.42)	0.712		
Histology	No Squamous	0.63 (0.40-1.01)	0.058	0.87 (0.52-1.46)	0.594
TNM	III	1.30 (0.76-2.22)	0.339		
Brain	NO	1.30 (0.75-2.27)	0.346		
Bone	NO	1.33 (0.85-2.09)	0.213		
Liver	NO	1.47 (0.79-2.71)	0.224		
Line	≥Second line	0.41 (0.27-0.64)	<0.001	0.35 (0.21-0.58)	<0.001
Strategy	Combination	0.83 (0.34-2.06)	0.689		
GC	NO	0.93 (0.60-1.42)	0.723		
ATB	NO	1.36 (0.83-2.22)	0.222		
PPIs	NO	0.76 (0.48-1.21)	0.250		
RT	NO	0.67 (0.43-1.05)	0.079	0.47 (0.28-0.77)	0.003

### 3.3 Subgroup analysis on the influence of GC timing on ICIs treatment outcomes in NSCLC

**Figure 3:**
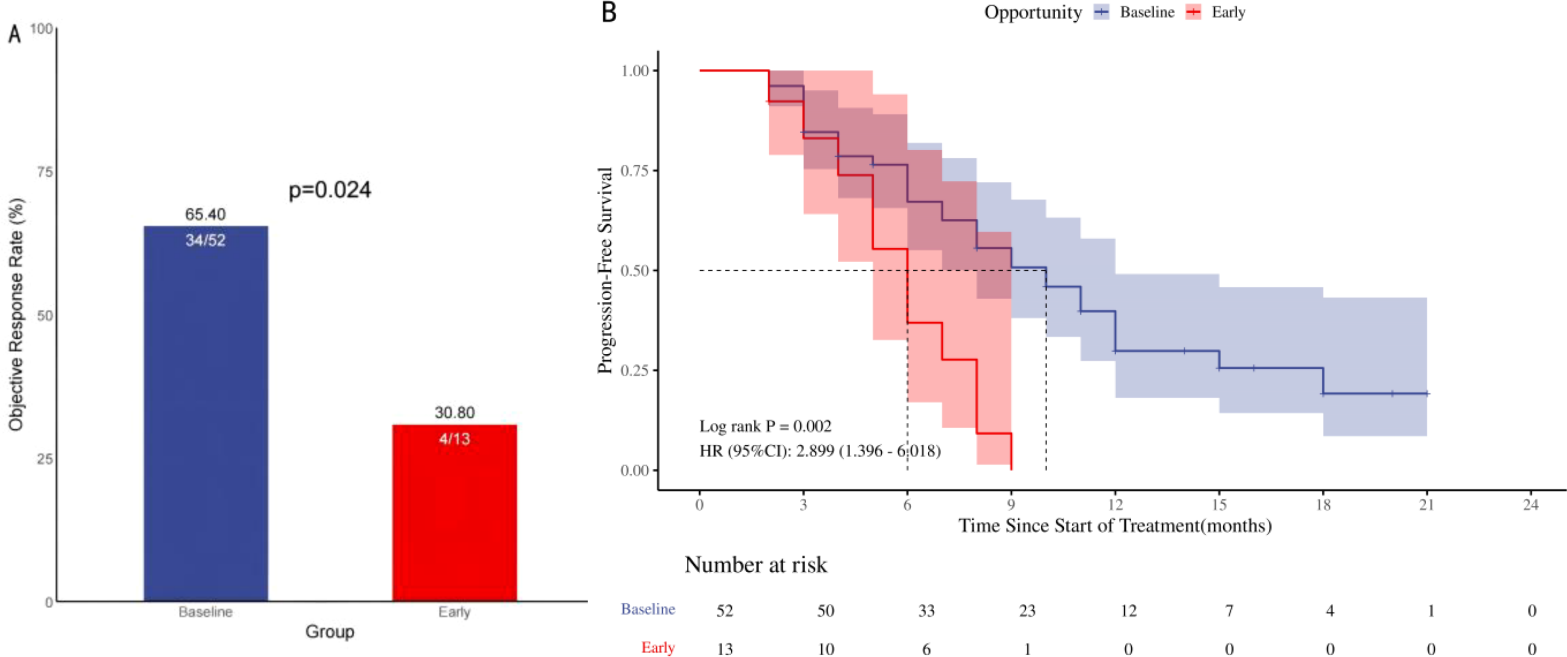
ORR and PFS in 65 patients with advanced NSCLC treated with ICIs in the GC group. Objective remission rate (**A**; ORR), progression-free survival (**B**; PFS), ORR and PFS in NSCLC patients treated with ICIs: Comparison of Baseline Treatment and Early Treatment with ≥ 10 mg/d prednisone.

Patients with baseline GC use exhibited a higher ORR compared to early GC use during ICIs treatment (65.4% vs 30.8%, p=0.024), as depicted in **Figure 3A**. In the univariate analysis, a substantial positive association was identified between the administration of GC at baseline and the ORR [HR=4.25, 95% CI (1.15-15.74), p=0.030]; **Figure 3A**. The multivariate analysis established that the use of GC at baseline was a significant and independent predictor of a favorable prognosis regarding the ORR in patients [HR=21.85, 95% CI (2.28-209.01), p=0.007]; **Table 4**. Higher ORR was significantly linked to squamous cell carcinoma and no brain metastases, but not to GC use reasons or other treatments like antibiotics and PPIs.

**Table 4:** Univariate and multifactorial analysis of ORR in 65 patients with advanced NSCLC treated with ICIs.

Characteristics	Reference	Univariate *HR* (95% *CI*)	p	Multivariate *HR* (95% *CI*)	p
Age	≤65	1.38 (0.50-3.78)	0.535			
Gender	Female	4.95 (1.56-15.69)	0.007	1.28 (0.20-8.18)	0.793
Smoke	NO	3.15 (1.13-8.81)	0.029	1.56 (0.28-8.59)	0.607	
ECOG	≤1	0.93 (0.35-2.49)	0.883			
Histology	No Squamous	9.09 (1.88-44.03)	0.006	16.63 (1.31-210.60)	0.030	
TNM	III	0.56 (0.17-1.85)	0.339			
Brain	NO	0.24 (0.06-0.87)	0.030	0.21 (0.05-0.98)	0.046	
Bone	NO	0.76 (0.27-2.10)	0.591			
Liver	NO	0.87 (0.21-3.60)	0.849			
Line	≥Second line	3.00 (1.01-8.91)	0.048	2.56 (0.66-9.86)	0.173	
Strategy	Combination	0.44 (0.07-2.86)	0.393			
Opportunity	Before	4.25 (1.15-15.74)	0.030	21.85 (2.28-209.01)	0.007	
Time	≤1Week	0.52 (0.19-1.42)	0.202			
Usedose	10-37.5 mg	1.09 (0.34-3.52)	0.890			
Totaldose	≤70mg	0.69 (0.25-1.86)	0.461			
Reason	NO-tumor causes	0.15 (0.01-1.56)	0.112			
		1.00 (0.08-12.56)	1.000			
		1.43 (0.21-9.54)	0.713			
ATB	NO	1.23 (0.43-3.58)	0.697			
PPIs	NO	1.18 (0.43-3.20)	0.749			
RT	NO	0.91 (0.34-2.46)	0.851			

A notable disparity in PFS was identified upon comparison of the two study groups [HR=2.90, 95% CI (1.40-6.02), p=0.002]; **Figure 3B**. The two survival curves exhibited clear separation, signifying a marked difference in survival outcomes between the groups. Univariate analysis showed that the baseline use of GC [HR = 0.34,95% CI (0.17-0.72), p = 0.004)], second-line and above treatment lines [HR = 0.45,95% CI (0.24-0.84), p = 0.012] was significantly positively correlated with PFS. Multivariable analysis indicated that baseline GC use [HR=0.37, 95% CI (0.17- 0.78), p=0.009] and treatment lines in the second line and above [HR=0.40, 95% CI (0.20- 0.81), p=0.011]; **Table 5** were significant prognostic factors for PFS.

**Table 5:** Univariate and univariate analysis of PFS in 65 patients with advanced NSCLC treated with ICIs.

Characteristics	Reference	Univariate *HR* (95% *CI*)	p	Multivariate *HR* (95%*CI*)	p
Age	≤65	0.77 (0.40-1.49)	0.442		
Gender	Female	0.48 (0.26-0.89)	0.020	0.79 (0.40-1.57)	0.503
Smoke	NO	0.73 (0.40-1.33)	0.303		
ECOG	≤1	0.75 (0.41-1.37)	0.348		
Histology	No Squamous	0.41 (0.18-0.93)	0.032	0.75 (0.28-2.00)	0.568
TNM	III	1.98 (0.83-4.68)	0.122		
Brain	NO	1.41 (0.69-2.80)	0.349		
Bone	NO	1.37 (0.74-2.51)	0.314		
Liver	NO	1.66 (0.79-3.48)	0.182		
Line	≥Second line	0.45 (0.24-0.84)	0.012	0.40 (0.20-0.81)	0.011
Strategy	Combination	1.62 (0.49-5.30)	0.426		
Opportunity	Before	0.34 (0.17-0.72)	0.004	0.37 (0.17-0.78)	0.009
Time	≤1Week	0.91 (0.50-1.68)	0.773		
Usedose	10-37.5 mg	0.74 (0.35-1.55)	0.425		
Totaldose	≤70mg	0.90 (0.49-1.66)	0.739		
Reason	NO-tumor causes	2.03 (0.56-7.44)	0.284		
		0.89 (0.09-8.68)	0.922		
		1.17 (0.35-3.87)	0.796		
ATB	NO	0.97 (0.50-1.89)	0.925		
PPIs	NO	0.66 (0.35-1.20)	0.196		
RT	NO	0.87 (0.47-1.60)	0.654		

### 3.4 NEUT%/(CD4+/CD8+) association with GC timing in ICIs treatment

**Figure 4:**
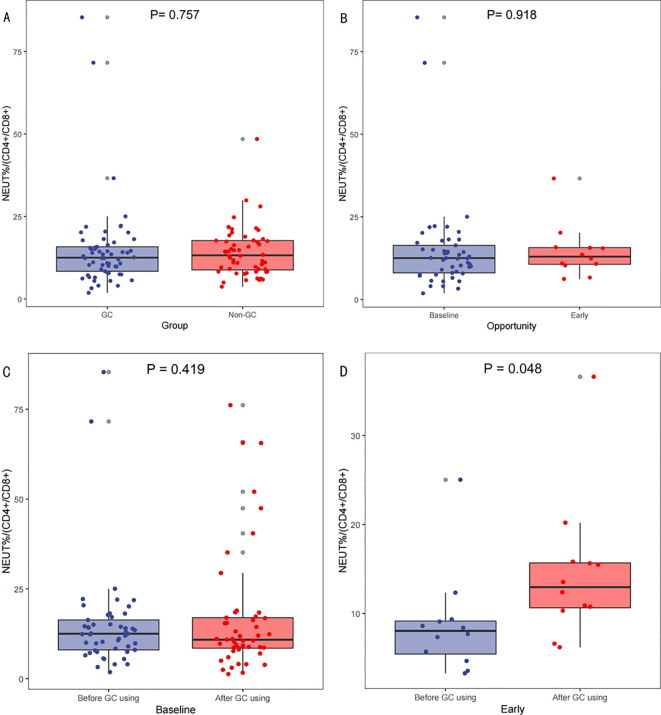
Variations in NEUT%/(CD4+/CD8+) across different scenarios. Comparison of neutrophil percentage to helper/suppressor T-lymphocyte ratio (NEUT%/(CD4+/CD8+))Before Treatment Between GC and Non-GC Groups **(A)**, Comparison of NEUT%/(CD4+/CD8+) at Baseline Versus Early GC Use **(B)**; and comparisons of the NEUT%/(CD4+/CD8+) levels before and after GC use at baseline **(C)** and early **(D)**.

Analysis of blood samples from 118 patients with complete blood information collected from 130 patients revealed no significant differences in the NEUT%/(CD4+/CD8+) between the GC and non-GC groups prior to treatment (p=0.757);**Figure 4A**. No differences were observed in NEUT%/(CD4+/CD8+) levels before baseline and early GC administration, p=0.918;**Figure 4B**. The paired-sample t-test showed no significant changes in NEUT%/(CD4+/CD8+) ratios pre- and post-baseline GC administration (p=0.419); **Figure 4C**, but there was a statistically significant difference in NEUT%/(CD4+/CD8+) levels before and after early GC use (p=0.048); **Figure 4D**. Lower levels of the NEUT%/(CD4+/CD8+) indicate a stable state of immune cell populations within patients and may thus respond better to ICIs therapy. This suggests that the timing of GC use, which influences the efficacy of ICIs, may be associated with the relative proportions and balance of immune cell populations.

## 4 Discussion

GC modulate immunity by inducing cell apoptosis, affecting differentiation, cytokine dynamics, cell migration, and clonal growth (14, 15). The efficacy of ICIs therapy may be reduced in patients who receive GC treatment at the onset of ICIs therapy (18, 25–27). Numerous studies have explored the effects of GC use in NSCLC patients undergoing ICIs therapy, focusing on factors such as indications, treatment duration, timing, and dosage (12, 19, 28).

In this study, GC use appeared to provide benefits, improving the ORR in patients receiving ICIs treatment, although the effect on PFS was not statistically significant. Among the 130 patients subjected to PSM, those who received GC exhibited a higher ORR, with no statistically significant difference in PFS between the two cohorts. Subgroup analyses on GC timing showed that patients starting GC at baseline had a significantly higher ORR and prolonged PFS. This suggests that the observed positive effect was primarily associated with the timing of GC administration, particularly when GC administration was initiated prior to the initiation of treatment, rather than the duration of GC use.

A meta-analysis indicates that patients receiving GC are at an elevated risk for disease progression and mortality (29). Current research mainly classifies GC indications as tumor-related or non-tumor-related, but determining whether GC directly causes poor prognosis or merely serves as a marker remains challenging (18, 20, 30). Some studies found no significant survival difference with GC use after adjusting for indications, suggesting that GC dose and timing may also influence prognosis (6–10, 28). The KEYNOTE-407 study found similar survival outcomes for patients with and without GC; however, it did not compare factors like GC timing or account for differences between preparations (31). GC at ≥ 10 mg/day prednisone equivalent significantly enhanced ICIs efficacy in managing irAEs. After adjusting for irAEs, timing also significantly influenced the outcome (30, 32, 33). Studies have shown that GC use within 28 days of initiating ICIs is associated with poor DCR, PFS, and OS, likely due to the regulation of peripheral blood immune cells (26). However, these studies did not control for factors like indications and PD-L1, and the time frame was too brief. Patients who initiated GC within two months of ICIs treatment showed extended PFS, regardless of clinical indications, consistent with this study’s findings (19). GC use within the first two months of ICIs treatment significantly prolonged PFS, with patients showing late responses (beyond six months) having better prognosis (29). These findings suggest that GC’s effect on ICIs efficacy is time-dependent and may be beneficial.

This study demonstrated that baseline or early GC use for ICIs treatment was strongly associated with better outcomes in patients with advanced NSCLC. ORR and PFS were significantly improved in patients treated with GC at baseline. This potential positive impact is closely related to the timing of GC use. Under certain conditions, GC administration does not reduce ICIs efficacy but may enhance the treatment response. This effect may be short-term and have limited impact on long-term survival. Possible explanations include the relative imbalance of the patient’s immune microenvironment at the start of treatment, with GC use contributing to immune cell activation and the initiation of anti-tumor responses, thereby alleviating symptoms and complications (34). The use of GC as chemotherapy pretreatment suggests that the patient is in good overall condition and that immunotherapy may have a synergistic effect with chemotherapy, mitigating some of GC’s negative effects (35). Notably, GC use in this study was limited to 30 days before and after the initial ICIs administration, and patients who received GC for irAEs may not have been sufficiently included (31). In this study, by fully including patients’ clinical information, especially concomitant treatments such as antibiotics and proton pump inhibitors, and adjusting for potential confounders by methods such as PSM, greatly reduced the confounding bias of the cause of tumour or other concomitant treatments on the use of GC (28). Variations in the definition of GC use duration may lead to differences in patient population delineation, potentially resulting in divergent study conclusions, suggesting that the biological effects of GC may be time-dependent.

The neutrophil-to-lymphocyte ratio (NLR) influences the effect of GC on OS in patients receiving ICIs treatment (36). Recognizing the variability in immune cell counts across individuals, the NEUT%/(CD4+/CD8+) ratio provides a more comprehensive depiction of the relative proportions and homeostatic balance among immune cell subsets. This study found that patients with lower levels of NEUT%/(CD4+/CD8+) appeared to respond better to ICIs treatment. The variation in NEUT%/(CD4+/CD8+) may be correlated with the timing of GC administration affecting patient prognosis, but whether this is its mechanism of action requires further research for validation.

No significant differences in ORR and PFS were observed between low and high doses of GC in this study. This finding may be attributed to the idea that the impact of GC on ICIs is less dose-dependent, with the timing being more influential in modulating patients’ immune status to produce a significant effect (37). The choice of dose cutoff values in this study, along with the variability in endogenous GC levels among patients, may also influence the results (38).

This study is limited by its single-center, real-world, retrospective design, making causal relationships difficult to determine. Missing PD-L1 expression data may introduce statistical bias and restrict the analysis of GC use timing (39). Larger-scale real-world or prospective studies are needed. PFS can mitigate bias from confounding factors and subsequent treatment differences; however, the long-term effects of GC on immunotherapy may not correlate as strongly with OS. In this study, the total daily dose of GC administered within 30 days before and after ICIs treatment was recorded. Given the frequent adjustments in GC dose (e.g., gradual reduction), the time frame for ICIs patients requiring GC treatment may not be sufficiently comprehensive. This study employs the PSM method to control confounding factors and achieve post-randomization, given the difficulty of conducting prospective studies. However, this approach may lead to population aggregation and a reduced sample size.

In summary, some theories worry that GC may have a negative impact on ICIs treatment. In fact, baseline or early GC use will enhance the efficacy of immunotherapy, which is closely related to the timing of GC use. The levels of NEUT %/(CD4 +/CD8 +) were different in different timing of GC use. GC should not be reduced or discontinued in the presence of clear clinical indications. Future research should focus on elucidating the specific mechanisms by which GC affects ICIs efficacy and optimizing GC use strategies to enhance immunotherapy outcomes.

## Data Availability

The datasets presented in this study can be found in online repositories. The names of the repository/repositories and accession number(s) can be found in the article/**Supplementary Material**.
