# Supplementary material for: The effect of baseline versus early glucocorticoid use on immune checkpoint inhibitor efficacy in patients with advanced NSCLC
Source: Front Oncol. 2025 Jan 23;15:1533556. doi: 10.3389/fonc.2025.1533556 (PMC11798793; doi:10.3389/fonc.2025.1533556)
Supplement: Supplementary file 1 [file DataSheet1.pdf]

Supplementary Material

Comparison of General Clinical Data between Patients Using GC before and after PSM
There was a balanced distribution between the baseline characteristics before and after PSM. Analysis of clinical outcomes of 286 patients with advanced NSCLC treated with ICIs before PSM revealed that patients with GC use had a higher ORR, and no significant difference in PFS was observed between the two groups. On subgroup analysis of the timing of GC use, baseline GC use had a higher ORR, longer PFS. This is consistent with the results derived from analysing the randomized cohort after PSM.

Supplementary Table 1 Clinical Characteristics of 286 Patients with Advanced NSCLC Receiving ICIs before PSM [n (%)]

Characteristics	Classification	Total (n = 286)	Non-GC (n=85)	GC (n=201)	χ^2	p
Gender						
	Female	56 (19.58)	19 (22.35)	37 (18.41)	0.59	0.442
	Male	230(80.42)	66 (77.65)	164(81.59)		
Age						
	≤65	159(55.59)	42 (49.41)	117(58.21)	1.87	0.171
	＞65	127(44.41)	43 (50.59)	84 (41.79)		
Smoke						
	NO	94 (32.87)	27 (31.76)	67 (33.33)	0.07	0.796
	Yes	192 (67.13)	58 (68.24)	134(66.67)		
ECOG						
	≤1	163(56.99)	41 (48.24)	122(60.70)	3.78	0.052
	＞1	123(43.01)	44 (51.76)	79 (39.30)		
Histology						
	No Squamous	162(56.64)	46 (54.12)	116(57.71)	0.31	0.575
	Squamous	124(43.36)	39 (45.88)	85 (42.29)		
TNM						
	III	85 (29.72)	22 (25.88)	63 (31.34)	0.85	0.356
	IV	201(70.28)	63 (74.12)	138(68.66)		
PD-L1						
	<1	28 (9.79)	10 (11.76)	18 (8.96)	0.58	0.902
	1-49	20 (6.99)	6 (7.06)	14 (6.97)		
	＞50	22 (7.69)	6 (7.06)	16 (7.96)		
	Unknown	216(75.52)	63 (74.12)	153(76.12)		
Brain						
	NO	237(82.87)	70 (82.35)	167(83.08)	0.02	0.881
	Yes	49 (17.13)	15 (17.65)	34 (16.92)		
Bone						
	NO	207(72.38)	61 (71.76)	146(72.64)	0.02	0.880
	Yes	79 (27.62)	24 (28.24)	55 (27.36)		
Liver						

Characteristics	Classification	Total (n = 286)	Non-GC (n=85)	GC (n=201)	χ²	p
	NO	263(91.96)	74 (87.06)	189(94.03)	3.93	0.048
	Yes	23 (8.04)	11 (12.94)	12 (5.97)		
Strategy						
	Combination	260(90.91)	64 (75.29)	196(97.51)	35.68	<.001
	Single	26 (9.09)	21 (24.71)	5 (2.49)		
Line						
	≥Second line	76 (26.67)	34 (40.00)	42 (21.00)	11.01	<.001
	First line	209(73.33)	51 (60.00)	158(79.00)		

Supplementary Table 2: Comparison of General Clinical Data between Patients Using GC before and after PSM [n (%)

Characteristics		Total (n = 201)	ahead (n= 150)	empress (n=51)	p	Total (n =65)	ahead (n= 52)	empress (n=13)	p
Gender									
	Female	37 (18.41)	29 (19.33)	8 (15.69)	0.562	19 (29.23)	14 (26.92)	5 (38.46)	0.633
	Male	164 (81.59)	121 (80.67)	43 (84.31)		46 (70.77)	38 (73.08)	8 (61.54)	
Age									
	≤65	117 (58.21)	90 (60.00)	27 (52.94)	0.377	38 (58.46)	31 (59.62)	7 (53.85)	0.706
	＞65	84 (41.79)	60 (40.00)	24 (47.06)		27 (41.54)	21 (40.38)	6 (46.15)	
Smoke									
	NO	67 (33.33)	54 (36.00)	13 (25.49)	0.169	28 (43.08)	23 (44.23)	5 (38.46)	0.707
	Yes	134 (66.67)	96 (64.00)	38 (74.51)		37 (56.92)	29 (55.77)	8 (61.54)	
ECOG									
	≤1	122 (60.70)	89 (59.33)	33 (64.71)	0.497	32 (49.23)	24 (46.15)	8 (61.54)	0.321
	＞1	79 (39.30)	61 (40.67)	18 (35.29)		33 (50.77)	28 (53.85)	5 (38.46)	
Histology									
	No Squamous	116 (57.71)	91 (60.67)	25 (49.02)	0.146	47 (72.31)	39 (75.00)	8 (61.54)	0.533
	Squamous	85 (42.29)	59 (39.33)	26 (50.98)		18 (27.69)	13 (25.00)	5 (38.46)	
TNM									
	III	63 (31.34)	45 (30.00)	18 (35.29)	0.481	16 (24.62)	10 (19.23)	6 (46.15)	0.098
	IV	138 (68.66)	105 (70.00)	33 (64.71)		49 (75.38)	4 (80.77)	7 (53.85)	
PD-L1									
	<1	18 (8.96)	12 (8.00)	6 (11.76)	0.050	8 (12.31)	5 (9.62)	3 (23.08)	0.218
	1-49	14 (6.97)	10 (6.67)	4 (7.84)		5 (7.69)	5 (9.62)	0 (0.00)	
	＞50	16 (7.96)	16 (10.67)	0 (0.00)		7 (10.77)	7 (13.46)	0 (0.00)	
	Unknown	153 (76.12)	112 (74.67)	41 (80.39)		45 (69.23)	35 (67.31)	10 (76.92)	
Brain									
	NO	167 (83.08)	121 (80.67)	46 (90.20)	0.117	52 (80.00)	39 (75.00)	13 (100.0)	0.104
	Yes	34 (16.92)	29 (19.33)	5 (9.80)		13 (20.00)	13 (25.00)	0 (0.00)	

Characteristics		Total (n = 201)	ahead (n= 150)	empress (n=51)	p	Total (n =65)	ahead (n= 52)	empress (n=13)	p
Bone									
	NO	146 (72.64)	109 (72.67)	37 (72.55)	0.987	41 (63.08)	33 (63.46)	8 (61.54)	1.000
	Yes	55 (27.36)	41 (27.33)	14 (27.45)		24 (36.92)	19 (36.54)	5 (38.46)	
Liver									
	NO	189 (94.03)	140 (93.33)	49 (96.08)	0.709	56 (86.15)	45 (86.54)	11 (84.62)	1.000
	Yes	12 (5.97)	10 (6.67)	2 (3.92)		9 (13.85)	7 (13.46)	2 (15.38)	
Strategy									
	Combination	196 (97.51)	146 (97.33)	50 (98.04)	1.000	60 (92.31)	48 (92.31)	12 (92.31)	1.000
	Single	5 (2.49)	4 (2.67)	1 (1.96)		5 (7.69)	4 (7.69)	1 (7.69)	
Line									
	≥Second line	42 (20.90)	27 (18.00)	15 (29.41)	0.083	20 (30.77)	15 (28.85)	5 (38.46)	0.737
	First line	159 (79.10)	123 (82.00)	36 (70.59)		45 (69.23)	37 (71.15)	8 (61.54)	

Prognostic Outcomes in 286 Patients with Advanced NSCLC Treated with ICIs Prior to PSM

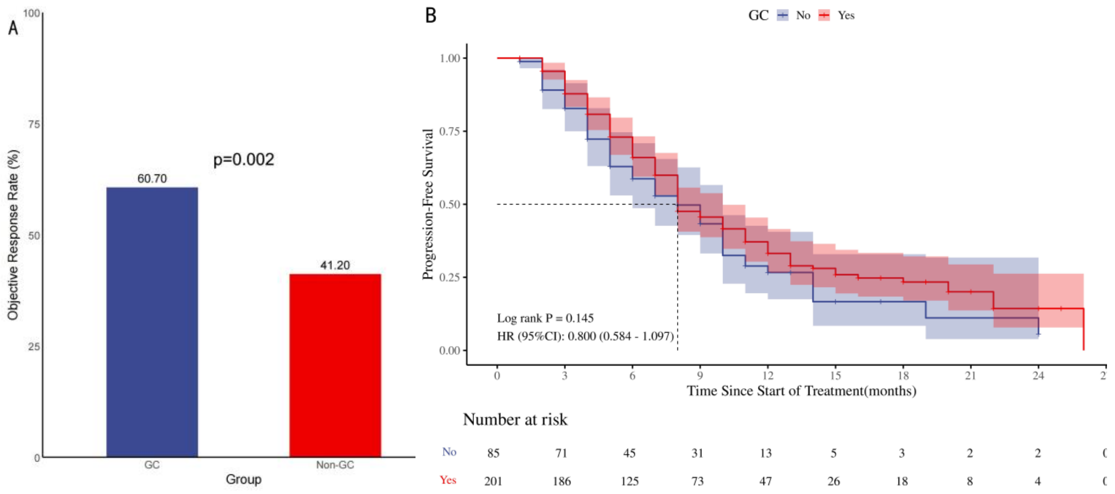

Supplementary Figure 1 ORR and PFS in 286 patients with advanced NSCLC treated with ICIs Supplementary Figure 1 Objective remission rate (A; ORR), progression-free survival (B; PFS), ORR and PFS in NSCLC patients treated with none or <10 mg/d prednisone versus those treated with ≥10 mg/d prednisone

Pre-PSM Subgroup Analysis of 201 Patients in the GC Group Focusing on the Timing of GC Administration

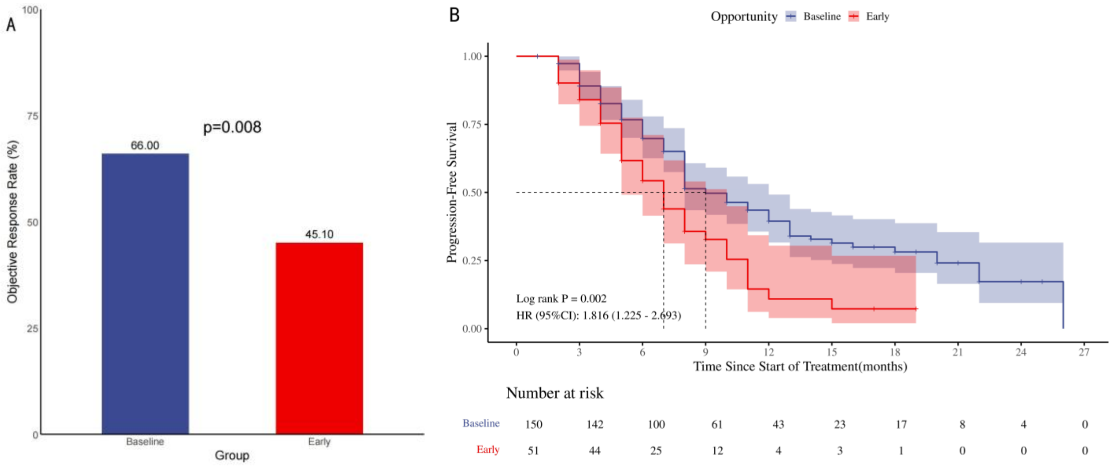

Supplementary Figure 2 ORR and PFS of 201 NSCLC patients treated with ICIs in the GC group
Supplementary Figure 2 Objective remission rate (A; ORR), progression-free survival (B; PFS), ORR and PFS in NSCLC patients treated with ICIs at baseline compared to those treated with ⩾10 mg/d prednisone prior

Data Availability Statement:

The data supporting the findings of this study are available in Figshare at **https://doi.org/10.6084/m9.figshare.28028879.v1** (Reference Number: 28028879).
